# Aicardi–Goutières syndrome with *SAMHD1* deficiency can be diagnosed by unscheduled DNA synthesis test

**DOI:** 10.3389/fped.2022.1048002

**Published:** 2022-11-04

**Authors:** Chikako Senju, Yuka Nakazawa, Mayuko Shimada, Dai Iwata, Michiko Matsuse, Katsumi Tanaka, Yasushi Miyazaki, Shinichi Moriwaki, Norisato Mitsutake, Tomoo Ogi

**Affiliations:** ^1^ Department of Hematology, Atomic Bomb Disease Institute, Nagasaki University Graduate School of Biomedical Sciences, Nagasaki, Japan; ^2^ Department of Plastic and Reconstructive Surgery, Nagasaki University Graduate School of Biomedical Sciences, Nagasaki, Japan; ^3^ Department of Genetics, Research Institute of Environmental Medicine, Nagoya University, Nagoya, Japan; ^4^ Department of Human Genetics and Molecular Biology, Graduate School of Medicine, Nagoya University, Nagoya, Japan; ^5^ Department of Genome Repair, Atomic Bomb Disease Institute, Nagasaki University, Nagasaki, Japan; ^6^ Department of Radiation Medical Sciences, Atomic Bomb Disease Institute, Nagasaki University, Nagasaki, Japan; ^7^ Department of Dermatology, Osaka Medical and Pharmaceutical University, Takatsuki, Japan

**Keywords:** Aicardi-Goutières syndrome (AGS), SAMHD1, unscheduled DNA synthesis (UDS), cockayne syndrome (CS), transcription coupled repair (TCR)

## Abstract

Aicardi–Goutières syndrome (AGS) is a rare genetic disorder characterised by progressive encephalopathy, involving microcephaly, intracranial calcification, and cerebrospinal fluid lymphocytosis with increased interferon-α concentrations. The clinical features of AGS overlap with fetal cerebral anomalies caused by congenital infections, such as TORCH (toxoplasmosis, other, rubella, cytomegalovirus, and herpes), or with those of other genetic disorders showing neonatal microcephaly, including Cockayne syndrome (CS) with transcription-coupled DNA repair deficiency, and Seckel syndrome (SS) showing aberrant cell-cycle checkpoint signaling. Therefore, a differential diagnosis to confirm the genetic cause or a proof of infection should be considered. In this report, we describe an individual who showed primordial dwarfism and encephalopathy, and whose initial diagnosis was CS. First, we conducted conventional DNA repair proficiency tests for the patient derived fibroblast cells. Transcription-coupled nucleotide excision repair (TC-NER) activity, which is mostly compromised in CS cases, was slightly reduced in the patient's cells. However, unscheduled DNA synthesis (UDS) was significantly diminished. These cellular traits were inconsistent with the diagnosis of CS. We further performed whole exome sequencing for the case and identified a compound heterozygous loss-of-function variants in the *SAMHD1* gene, mutations in which are known to cause AGS. As *SAMHD1* encodes deoxyribonucleoside triphosphate triphosphohydrolase, we reasoned that the deoxyribonucleoside triphosphate (dNTP) pool size in the patient's cells was elevated, and the labeling efficiency of UDS-test was hindered due to the reduced concentration of phosphorylated ethynyl deoxyuridine (EdU), a nucleoside analogue used for the assay. In conclusion, UDS assay may be a useful diagnostic tool to distinguish between AGS with *SAMHD1* mutations and other related diseases.

## Introduction

Aicardi–Goutières syndrome (AGS) is a rare neurodegenerative autoimmune disorder, which was first described by Aicardi and Goutieres in 1984 (1, 2). Initially, AGS was thought to be caused by an *in utero* infection on the basis of clinical similarities to known congenital infectious diseases, such as TORCH (toxoplasmosis, other, rubella, cytomegalovirus, and herpes) infection (3). However, AGS turned out to be a hereditary disease due to a malfunction of the antiviral interferon response system. To date, pathogenic variants associated with typical AGS have been reported in nine genes (*TREX1, RNASEH2A, RNASEH2B, RNASEH2C, SAMHD1, ADAR1*, *IFIH1*, *LSM11* and *RNU7–1*) (4–11). The common symptoms of AGS include psychomotor impairment, progressive microcephaly, basal ganglia calcification, spasticity, and dystonia. Patients with AGS also develop systemic lupus erythematosus (SLE)-associated autoantibodies (12, 13). They also show erythema with photosensitivity (14), which are common characteristic of infantile SLE, and their symptoms resemble those of Cockayne syndrome (CS). CS is an autosomal recessive neurodegenerative disorder characterised by photosensitivity, microcephaly, progressive growth failure, intellectual disability, and neuropsychiatric symptoms (15). CS is associated with a dysfunction in transcription-coupled nucleotide excision repair (TC-NER), which is a sub-pathway of nucleotide excision repair (NER) (16–18). TC-NER removes ultraviolet (UV)-induced photolesions and bulky DNA adducts preferentially from actively transcribed regions.

Because of an overlap of clinical manifestations between AGS and CS, their differential diagnosis is often challenging. In this report, we describe a patient with an initial diagnosis of CS, which eventually turned out to be AGS with mutations in the *SAMHD1* gene. From our regular DNA repair proficiency tests performed for typical-CS cases, the patient had been initially thought to be an NER-deficient disease (19), but no obvious aberration was detected in known NER genes (20, 21). Whole exome sequencing (WES) identified biallelic mutations in the *SAMHD1* gene, which encodes an antiviral protein that acts as a deoxyribonucleoside triphosphate triphosphohydrolase (dNTPase) (6, 22). The dNTPase activity of SAMHD1 decreases the deoxyribonucleoside triphosphate (dNTP) pool and inhibits reverse transcription of the viral genome (23). Because of the increased dNTP pool size in the patient's cells, the labeling efficiency of unscheduled DNA synthesis (UDS) test, which is usually used for the purpose of measuring NER capacity (19, 24, 25), was hindered. We examined the NER function of SAMHD1 and further discuss the differences between AGS and CS symptoms. The present findings suggest that UDS test is a useful tool for the differential diagnosis of AGS with mutations in *SAMHD1* and other related diseases such as CS.

## Materials and methods

### Cell culture

The following primary fibroblasts were used. 48BR was from a normal human subject (26). CS10LO was derived from a patient with CS complementation group B (CS-B). XP21BR was derived from a patient with xeroderma pigmentosum complementation group C (XP-C) (24). XP15BR was derived from a patient with xeroderma pigmentosum complementation group A (XP-A) (27). CS213NG was derived from the present case. All fibroblasts were cultured in Dulbecco's modified Eagle's medium (Wako; Cat. No. 043–30085) supplemented with 10% fetal bovine serum (Thermo Fisher Scientific; Cat. No. 10437–028) and 1% penicillin-streptomycin (Wako; Cat. No. 168–23191).

### NER activity

#### Unscheduled DNA synthesis (UDS) and recovery of RNA synthesis (RRS) assays

NER activity was measured using unscheduled DNA synthesis (UDS) and recovery of RNA synthesis (RRS) assays. Details of the experimental procedures were described previously (23, 24). Normal 48BR, CS patient-derived CS10LO (CS-B), XP patient-derived XP21BR (XP-C) and XP15BR (XP-A), as well as CS213NG patient fibroblasts were plated into 96-well plastic dishes. In UDS assay, cells were irradiated with 20 J/m^2^UV (254 nm) and immediately incubated with 5 μM 5-ethynyl-2′-deoxyuridine (EdU, Thermo Fisher Scientific; Cat. No. A10044) for 4 h to measure non-S phase DNA synthesis activity associated with global genome repair (GGR), which is compromised in typical XP-patient cells. In RRS assay, cells were irradiated with UV (13 J/m^2^), followed by 18 h of recovery and subsequent 2 h incubation with a medium containing 5-ethynyluridine (EU, Thermo Fisher Scientific; Cat. No. E10345). This measures transcription recovery, activity of which is diminished in CS patient cells. After EdU- or EU-incorporation, the cells were fixed, conjugated with Alexa Fluor 488 azide (Thermo Fisher Scientific, A10266), and stained with DAPI (Dojindo; Cat. No. D523). Incorporated EdU or EU in each cell nucleus were measured using an automated image acquisition and processing system (Cellomics ArrayScan VTI; Thermo Scientific). As UDS assay measures EdU incorporation levels of non-S phase cells, we select an appropriate intensity cutoff level of EdU nuclear fluorescence to exclude high-intensity cells under S-phase DNA replication.

#### Lentivirus complementation assay

Experimental details of the production of lentivirus particles expressing the human *XPA*-*XPG* and *ERCC1* cDNAs have been described previously (19, 28, 29). Virus complementation experiments for UDS assay were performed using CS213NG cells. These cells were plated on 96-well plastic dishes and infected with the lentiviruses*.* The cells were subjected to UDS assay at 66–72 h after viral infection, followed by EdU-incorporation and detection.

### Immunoblotting

Cells were lysed in 2.5% sodium dodecyl sulfate sample buffer, followed by 5 min incubation at 96 °C. Equivalent amounts of protein samples were resolved with 5%–20% gradient sodium dodecyl sulfate-polyacrylamide gel electrophoresis gels, and blotted onto polyvinylidene difluoride membranes. The membranes were blocked with 10% skim-milk in TBS-T (0.05% Tween-20), followed by overnight incubation at 4 °C with anti-SAMHD1 antibodies (Cell Signaling Technology; Cat. No. 12,361S; source: rabbit) at a 1:250 dilution, or anti-*β*-actin antibodies (Santa Cruz Biotechnology; Clone C-4; Cat. No. sc-47,778; source: mouse) at a 1:1,000 dilution. After extensive washing with TBS-T (0.05% Tween-20), the membranes were incubated with secondary antibodies against anti-rabbit immunoglobulin G (Cell Signaling Technology; Cat. No. 7074) at a 1:500 dilution, or anti-mouse immunoglobulin G (Cell Signaling Technology; Cat. No. 7076) at a 1:1,000 dilution. The proteins were detected using an enhanced chemiluminescence system (Luminescent Image Ana-Lyzer LAS-3000; Fujifilm).

### Measurement of cyclobutane pyrimidine dimers and 6–4 photoproducts by enzyme-linked immunosorbent assay

DNA repair activity was measured by residual UV-photolesions. Cells were plated in 10 cm dishes and cultured to reach confluence. The cells were then 15 J/m^2^ irradiated by UV, followed by incubation for different times (cyclobutane pyrimidine dimers [CPDs]: 24, 48, 72, and 96 h; 6–4 photoproducts [6–4PPs]: 1, 3, 6, and 24 h). After harvesting the cells, genomic DNA was extracted using a QIAamp DNA Mini Kit (Qiagen; Cat. No. 51306). Genomic DNA samples were prepared respectively at 0.2 μg/ml for CPD measurements and at 4.0 μg/ml for 6–4PP measurements. DNA samples were denatured at 100 °C for 10 min and placed on ice for 15 min. A 50 μl aliquot of the sample DNA solution was added to each well of a protamine sulfate-coated enzyme-linked immunosorbent assay plate (Cosmo Bio; Cat. No. NM-MA-P001) and completely dried by overnight incubation at 37 °C. Non-specific antibody-binding sites of the DNA samples were depleted by blocking with 2% fetal bovine serum in phosphate-buffered saline (PBS) at 150 μl/well. The DNA samples were then incubated with anti-CPD antibodies (TDM-2, Cosmo Bio; Cat. No. NMDND001) or with anti-6–4PPs antibodies (64M-2, Cosmo Bio; Cat. No. NMDND002), and both samples were diluted in PBS (1:1000) at 100 μl/well for 30 min at 37 °C. After extensive washing with PBS-T (0.05% Tween-20), the plates were incubated with horseradish peroxidase-conjugated rat-anti-mouse secondary antibodies (Cell Signaling Technology; Cat. No. 7076) diluted in PBS (1:1000) at 100 μl/well for 30 min at 37 °C. After five times wash with PBS-T (0.05% Tween-20) at 150 μl/well, 100 μl of tetramethylbenzidine peroxidase substrate solution (Sigma; Cat. No. T0440) was added to each well and incubated for 30 min at room temperature in the dark. The reaction was stopped with 100 μl of 0.5 M H_2_SO_4_. The fluorescence intensity was measured at 450 nm with an enzyme-linked immunosorbent assay plate reader (Multiskan FC; Thermo Scientific).

### Exome sequencing

To identify pathogenetic variants in the patient's genome, WES was conducted as described previously (28). DNA fragments were enriched by using the Agilent Sure Select Human All Exon Kit v.6 (Agilent, Santa Clara, CA, United States). The captured exonic genomic DNA fragments were sequenced on the Illumina Hiseq 2,500 platform (Illumina, San Diego CA, United States), with paired-end (PE) flow cells to obtain 100–150 base pair reads of 100–200 ×  exon coverage. The next-generation sequencing data were analysed using our standard exome pipeline. All of the variants were annotated with ANNOVAR (30) on the basis of GENCODE release 19 (GRCh37).

## Results

### Case report

The patient (CS213NG) was a boy born uneventfully at 40 weeks of gestation with a birth weight of 2452 g. At 3 years of age, his body weight was 8.6 kg (−2 standard deviations [SDs]), his height was 82 cm (−3SDs), and his head circumference was 45.7 cm (−2.4SDs). The patient showed psychomotor impairment and spastic paralysis of both lower extremities. A rash due to photosensitivity was observed on both cheeks. Calcification was noted in the bilateral basal ganglia on a head computed tomography scan. His symptoms resembled those of CS.

### NER activity in the patient's cells

Because the patient displayed CS-like clinical features, we initially measured NER activity in the patient's CS213NG fibroblasts. Global genome NER (GG-NER) removes damages throughout the genome, while TC-NER removes damages from actively transcribed genes. GG-NER and TC-NER activities can be measured by UDS (31) and RRS (32) assays, respectively.

**Figure 1:**
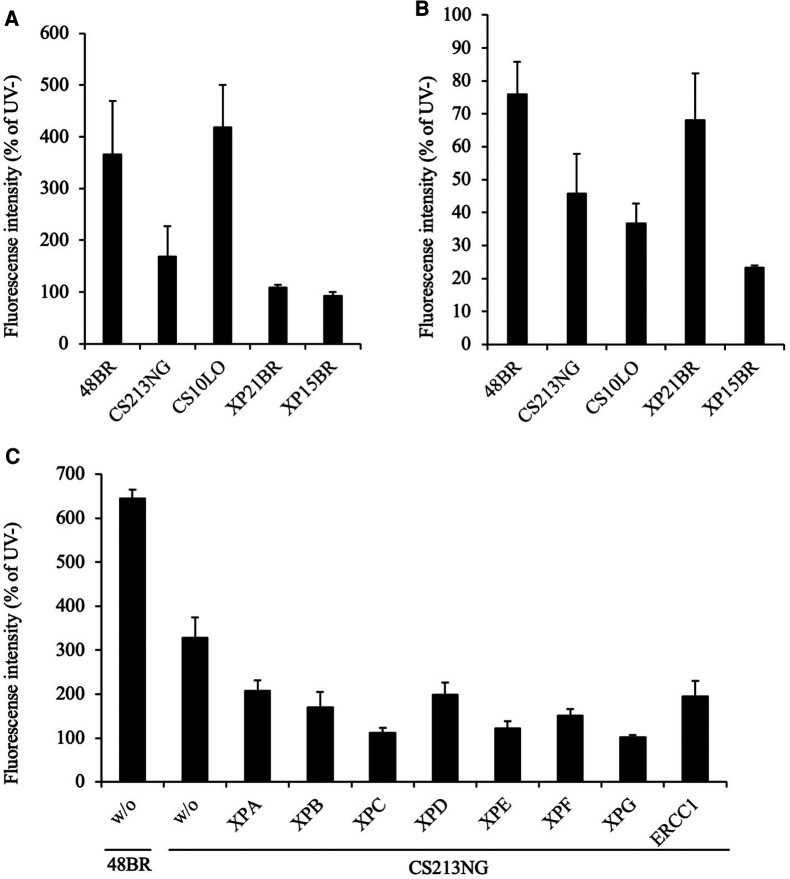
**Examination of NER activity by UDS and RRS assays.** (**A**) UDS activity after UV irradiation was impaired in CS213NG cells. 48BR (Normal), CS10LO (CS-B), XP21BR (XP-C), XP15BR (XP-A), and the patient-derived CS213NG cells were irradiated with 20 J/m^2^ UVC followed by UDS measurements (*p < 0.05, two-tailed homoscedastic *t*-test, 48BR vs. CS213NG). (**B**) Normal but slightly reduced RRS activity in CS213NG cells (*p < 0.05, 48BR vs. CS213NG; 13 J/m^2^ UVC irradiation). UDS and RRS were normalised to activity in non-irradiated cells. Error bars represent the standard deviation (SD) of the mean nuclear fluorescent intensity in triplicate samples (**A**, **B**). (**C**) UDS virus complementation assay (representative data). CS213NG cells were infected with lentiviruses expressing either of the *XPA*-*XPG* as well as *ERCC1* cDNAs. Error bars represent the standard deviation of four wells.

In UDS assay, normal 48BR and CS-B patient-derived CS10LO fibroblasts showed normal levels of EdU incorporation after UV irradiation and were proficient in GG-NER, while *XPC*-deficient XP21BR and *XPA*-deficient XP15BR fibroblasts showed no EdU incorporation due to the lack of GG-NER activity (Figure 1A). Unexpectedly, UV-induced EdU incorporation in CS213NG cells was significantly reduced compared with that in 48BR cells, which indicated that the patient's CS213NG cells had substantial UDS deficiency (Figure 1A). In RRS assay, CS10LO and XP15BR cells showed impaired TC-NER function, with decreased EU incorporation after UV-irradiation, while 48BR and XP21BR cells showed normal RRS levels (Figure 1B). Contrary to our expectation, RRS activity was only mildly decreased in the patient's CS213NG cells. These findings suggested that the patient did not have typical CS (Figure 1B).

On the basis of the reduced UDS activity, we anticipated that the patient carried pathogenic mutations either in the GG-NER related genes. We tested complementation of the UDS defects by infecting the patient's CS213NG cells with lentiviruses expressing one of the genes (cDNAs) associated with the entire NER pathway. Unexpectedly, neither gene complemented the UDS activity compared with 48BR cells. Therefore, NER-associated genes were not responsible for the disease (Figure 1C).

### Exome sequencing identifies loss of function variants in *SAMHD1*

**Figure 2:**
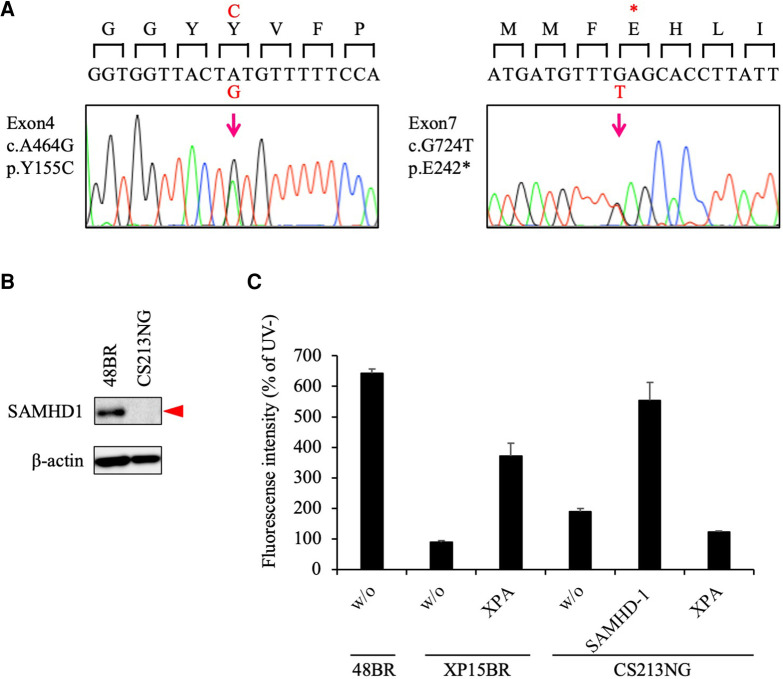
**Pathogenic variants identified in the *SAMHD1* gene.** (**A**) Sanger sequencing confirmed that CS213NG is compound heterozygous for the *SAMHD1* c.A464G and c.G724T. (**B**) Lack of the SAMHD1 protein expression in CS213NG cells. Bata-actin was a loading control. (**C**) Ectopic expression of the *SAMHD1* cDNA complemented the UDS defect of CS213NG cells. CS213NG and XP15BR cells were infected with lentiviruses expressing either of the *XPA* or *SAMHD1* cDNAs (representative data). Error bars represent the standard deviation of four wells.

To identify disease causative variants carried in the patient, we then performed exome sequencing (WES). Under a recessive model of inheritance, we identified potentially pathogenic mutations in the *SAMHD1* gene, which is known to be associated with AGS. The identified variants were c.G724T in exon 7, resulting a truncation mutation, *p*. Glu242Ter (pathogenic - PVS1/PM2/PP3, based on the American College of Medical Genetics and Genomics (ACMG) and the Association for Molecular Pathology (AMP) guideline, [ACMG/AMP] 2015; no variant allele was found in gnomad v.2.1.1), and c.A464G in exon 4 leading to an amino acid substitution, *p*.Tyr155Cys (likely pathogenic - PS3 (33, 34)/PM1/PM2/PP3, based on the ACMG/AMP 2015; rs1306109528; 1 in ∼250 k alleles in gnomad v.2.1.1) (Supplementary Table S1); these mutations were confirmed by Sanger sequencing (Figure 2A).

As the identified variants have not yet been reported previously as any congenital disease-causing mutations, we next studied the consequences of the variants. While the SAMHD1 protein was detected at 72 kDa by immunoblotting in normal 48BR cells, this band was not detectable in the patient's CS213NG cells (Figure 2B). This result is consistent with previous reports describing the instability nature of the missense variant, *p*. Y155C, *in vitro* (34), or in leukemia patients' cells with this mutation (33).

We further performed a lentivirus complementation test. Overexpression of the wild-type *SAMHD1* cDNA in the patient's cells restored normal UDS (Figure 2C). These data indicated that the identified *SAMHD1* variants were causal for the patient's cellular phenotype and new pathogenic mutations. Therefore, we concluded that the patient did not have CS, but had AGS instead.

### Reduced EdU incorporation capacity of the patient's cells due to an increase in the dNTP pool size

**Figure 3:**
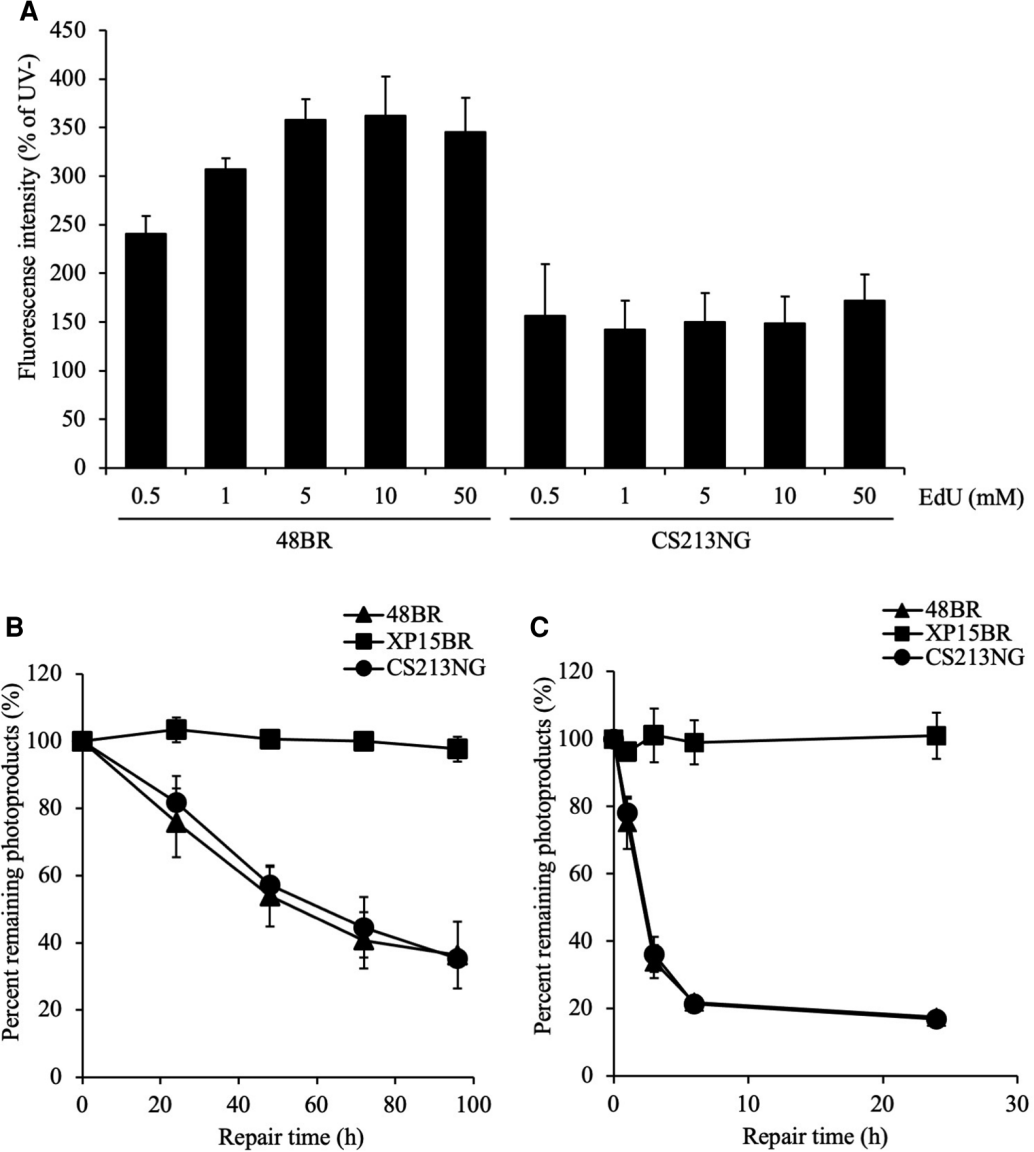
**Reduced UDS in CS213NG cells is associated with an increased dNTP pool size.** (**A**) A UDS assay was performed with various concentrations of EdU. 48BR and CS213NG cells were incubated with increasing EdU concentrations from 0.5 to 50 μM. Error bars represent the SD of means of nuclear fluorescent intensity in triplicate samples. (**B**, **C**) Normal DNA repair kinetics in CS213NG cells. Detection of CPD (**B**) and 6-4PP (**C**) damages. Cells were irradiated by UV at 15 J/m^2^ and incubated for various times to allow repair. The error bars represent the SD of triplicate experiments.

The *SAMHD1* gene encodes a major dNTPase, which depletes cellular dNTPs. Therefore, we deduced that the compromised UDS test of the *SAMHD1*-deficient CS213NG cells was due to an increase in the cellular dNTP pool size and an eventual reduction in the EdU labeling efficiency, rather than a lack of repair DNA synthesis associated with NER activity. To test this possibility, we performed further UDS tests of the patient's cells under various concentrations of EdU (Figure 3A). We have previously shown that the EdU incorporation efficiency is dependent on its concentration relative to endogenously synthesized thymine nucleotides in the nucleotide pool (24). In the normal 48BR cells, UDS activity was increased with high EdU concentrations and reached a plateau at 5 μM. While in the *SAMHD1*-deficient CS213NG cells, the UDS activity remained lowish at 0.5–50 μM of EdU. These data strongly suggest that the dNTP concentration in the *SAMHD1*-deficient AGS patient cells is much higher than that of normal cells. Collectively, the abnormal UDS result of CS213NG is a consequence of an increase of dNTP pool size rather than an NER defect.

### Normal DNA repair activity confirmed by CPD and 6–4pp repair assays

Considering that DNA repair activity is proficient in *SAMHD1*-deficient cells, we further assessed the NER function and repair kinetics of major photolesions, cyclobutane pyrimidine dimers (CPDs) and pyrimidine-pyrimidone 6–4 photoproducts (6–4PPs), in CS213NG cells (Figures 3B,C). While NER-deficient XP15BR cells exhibited no repair of CPDs and 6–4PPs, CS213NG cells displayed similar repair kinetics to those of normal 48BR cells, which indicates that CS213NG cells are proficient in NER. This result is consistent with a previous report describing a proficiency (maybe rather efficient) in DNA repair of major photolesions in *SAMHD*-deficient fibroblasts from AGS patients (35). Collectively, the disease-responsible gene in the patient CS213NG was *SAMHD1*. The lack of SAMHD1 protein apparently reduces UDS activity, but its NER function is proficient.

## Discussion

Prompt establishment of a definite diagnosis in patients with rare disorders is important from the point of view of the patient's quality of life. As summarised in Table 1, AGS and CS have various overlapping phenotypes, including basal ganglia calcification, intellectual disability, developmental delay, muscle stiffness, microsomia, microcephaly, and hepatosplenomegaly. While CS shows characteristic erythema after UV irradiation, AGS may have a clinical overlap with SLE resulting in a lupus-like rash. Because of these similar clinical manifestations, the differential diagnosis of CS and AGS, as well as other related disorders, is often difficult.

**Table 1:** Common clinical manifestations in Aicardi–Goutières syndrome and cockayne syndrome, and symptoms observed in the present patient (CS213NG).

Symptoms	CS213NG	Aicardi–Goutières syndrome	Cockayne syndrome
Basal ganglia calcification	+	+	+
Increased cerebrospinal fluid interferon-α	?	+	−
Intellectual disability	+	+	+
Developmental delay	+	+	+
Muscle stiffness (spasticity)	+	+	+
Microsomia	+	+	+
Microcephaly	+	+	+
Hepatosplenomegaly	?	+	+
Chilblains	?	+	−
Photosensitivity	+	+ (When combined with SLE)	+
Progeroid	−	−	+
Sensorineural hearing loss	?	±	+
Pigmentary retinopathy	−	−	+
GG-NER (UDS activity*)	Defective	Normal*	Normal
TC-NER (RRS activity)	Normal	Normal	Defective
CPD, 6-4PP repair	Normal	Normal	Normal

* UDS is defective when *SAMHD1* is compromised.

In the present case, CS213NG, the initial diagnosis based on the clinical appearance was CS. Most typical CS cases are caused by a deficiency of the *CSA* or *CSB* gene. Because the CSA/CSB protein complex roles in the initiation process of TC-NER, by ubiquitinating DNA damage stalled RNA polymerase IIo molecules, CSA/CSB-compromised patients cells display TC-NER deficiency (36). Therefore, laboratory-based CS clinical diagnoses rely on UDS and RRS tests to determine the cellular NER capacity. RRS-negative is a key endpoint of CS-patient cells compromised in the CSA/CSB protein complex. RRS-proficiency can eliminate the possibility of non-CS conditions, such as TORCH infections showing congenital intracranial calcification, and hereditary microcephalic disorders including Seckel syndrome. In addition, UDS-deficiency is observed in patients with specific minor mutations in the NER responsible *XPB*, *XPD*, and *XPG* genes; they display the combined features of CS and another genodermatosis, xeroderma pigmentosum (XP), termed XPCS. According to the circumstances, even typical CS cases with mutations in the *CSA* or *CSB* gene might display an intermediate RRS deficiency. In the present case, the RRS reduction was not significant enough to determine a TC-NER defect; however, the decrease of UDS was prominent. Therefore, we initially presumed that the patient falls into XPCS with mutations either in the GG-NER gene. Unexpectedly, the lentivirus complementation test revealed that neither of the *XPA*-*XPG* as well as *ERCC1* complemented the UDS defect of the patient's CS213NG cells. At this point, we anticipated a possibility that the patient may carry pathogenic variants in a gene for which CS-causing mutations have not yet been identified.

The use of next generation sequencing (NGS) has become popular for clinical genetics. We routinely perform whole exome sequencing (WES) for the diagnosis of uncharacterised genetic disorders. Although WES provides a rapid diagnosis compared to conventional cellular functional analyses, the overall diagnostic rate of rare diseases by WES is still insufficient in general clinical practice. This is particularly because of the low detection power of non-coding variants as well as the necessity of extra evaluations of identified variants of unknown significance (VUSs). For these reasons, in case of CS genetic tests, we ordinary rely on the target Sanger sequencing of the *CSA*/*CSB* genes. Because the cellular NER property of CS213NG was inconsistent with the diagnosis of CS, we further performed WES on the case to search for potentially pathogenic variants. The WES identified 18 candidate genes with homozygous or compound heterozygous mutations under a recessive model of inheritance (Supplementary Table S1). We focused on the *SAMHD1* gene as mutations in this gene are known to cause AGS, a disease with similar pathology to CS. According to the ACMG guideline, the variants identified in the *SAMHD1* gene were evaluated as a pathogenic stopgain mutation, *p*. E242X, and a likely-pathogenic amino acid substitution, *p*. Y155C. These WES evaluations were sufficient to suspect that the patient has AGS, but are still insufficient to provide a definite diagnosis; therefore, further experimental evaluation of variants was needed.

The identified *SAMHD1* variants were both proved to be loss-of-function pathogenic mutations because the SAMHD1 protein expression was absolutely absent and the UDS defect was complemented by the ectopic expression of the *SAMHD1* cDNA in the patient's cells. As *SAMHD1* encodes a major dNTPase, the reason for the UDS deficiency in the patient's cells was due to an increase of the dNTP pool size and, as a consequence, a decrease of the EdU labeling efficiency of DNA synthesis associated with NER. This deduction is intelligible for the explanation of *ΔSAMHD1* cellular phenotype; however, in terms of the use of UDS and RRS tests for the diagnosis of NER-deficient disorders such as CS and XP, the UDS-deficiency was unexpected. The current experience indicates that UDS test is also effective for a cytological examination of *SAMHD1*-deficient AGS cases. In conclusion, UDS and RRS assays are useful diagnostic tools to distinguish between AGS with *SAMHD1* mutations and other similar disorders.

## Data Availability

The raw data supporting the conclusions of this article will be made available by the authors, without undue reservation.
