# Supplementary material for: Aicardi–Goutières syndrome with SAMHD1 deficiency can be diagnosed by unscheduled DNA synthesis test
Source: Front Pediatr. 2022 Nov 4;10:1048002. doi: 10.3389/fped.2022.1048002 (PMC9673124; doi:10.3389/fped.2022.1048002)
Supplement: Supplementary file 1 [file Table1.docx]

Supplementary Material

**Table 1. Homozygous and compound heterozygous variants in the pathogenic candidate genes identified by exome sequencing of the CS213NG**

| **chr.** | **position** | **zygosity** | **gene** | **mutation type^*^** | **nucleotide change** | **amino-acid change** | **SIFT** |
| --- | --- | --- | --- | --- | --- | --- | --- |
| 1 | 148591241 | hom | *NBPF15* | ns | c.T1306A | p.L436M | 0.22 |
| 5 | 150521091 | hom | *ANXA6* | splicing |  |  | 0 |
| 9 | 101788220 | het | *COL15A1* | ns | c.C2015A | p.T672N | 0.5 |
| 9 | 101825336 | het | *COL15A1* | ns | c.A3596G | p.H1199R | 0.14 |
| 11 | 117314620 | het | *DSCAML1* | ns | c.A4024G | p.I1342V | 1 |
| 11 | 117321348 | het | *DSCAML1* | ns | c.G3805C | p.V1269L | 0.09 |
| 12 | 108956433 | hom | *ISCU* | ns | c.C35G | p.A12G | 0.15 |
| 15 | 42149638 | het | *SPTBN5* | ns | c.G8419A | p.E2807K | 0.07 |
| 15 | 42154408 | het | *SPTBN5* | ns | c.C7468T | p.R2490W | 0.01 |
| 16 | 75239282 | hom | *CTRB2* | ns | c.C445T | p.P149S | 0.04 |
| 17 | 78262109 | het | *RNF213* | ns | c.C757T | p.P253S | 0.04 |
| 17 | 78316961 | het | *RNF213* | ns | c.G6019A | p.D2007N | 0.28 |
| 19 | 43411772 | hom | *PSG6* | ns | c.G941A | p.R314Q | 0.2 |
| **20** | **35547895** | **het** | ***SAMHD1*** | **stopgain** | **c.G724T** | **p.E242*** | **0.16** |
| **20** | **35563477** | **het** | ***SAMHD1*** | **ns** | **c.A464G** | **p.Y155C** | **0** |
| 21 | 33887124 | hom | *EVA1C* | ns | c.C950T | p.A317V | 0 |
| 22 | 21337379 | hom | *LZTR1* | splicing |  |  | 0 |
| 22 | 38308003 | het | *MICALL1* | ns | c.G191A | p.R64H | 0.33 |
| 22 | 38336774 | het | *MICALL1* | ns | c.G2529T | p.K843N | 0.01 |
| X | 70147764 | hom | *SLC7A3* | ns | c.G927A | p.M309I | 0 |
| X | 135314139 | hom | *MAP7D3* | ns | c.G977A | p.G326D | 0.46 |
| X | 135430430 | hom | *ADGRG4* | ns | c.C4565T | p.A1522V | 0 |
| X | 153172097 | hom | *AVPR2* | ns | c.G1031A | p.R344Q | 0.44 |
| Y | 6736361 | hom | *AMELY* | ns | c.G290A | p.R97H | 0 |

*****ns/nonsynonymous SNV
